# Junior medical doctors’ decision making when using advance care directives to guide treatment for people with dementia: a cross-sectional vignette study

**DOI:** 10.1186/s12910-022-00811-x

**Published:** 2022-07-14

**Authors:** Amy Waller, Jamie Bryant, Alison Bowman, Ben P. White, Lindy Willmott, Robert Pickles, Carolyn Hullick, Emma Price, Anne Knight, Mary-Ann Ryall, Mathew Clapham, Rob Sanson-Fisher

**Affiliations:** 1 Health Behaviour Research Collaborative, College of Health, Medicine and Wellbeing, University of Newcastle, Callaghan, NSW 2308 Australia; 2 School of Medicine and Public Health, College of Health, Medicine and Wellbeing, University of Newcastle, Callaghan, NSW Australia; 3 Priority Research Centre for Health Behaviour, University of Newcastle, Callaghan, NSW Australia; 4 Hunter Medical Research Institute, New Lambton Heights, NSW Australia; 5 Australian Centre for Health Law Research, Queensland University of Technology, Brisbane, QLD Australia; 6 John Hunter Hospital, Hunter New England Local Health District, Newcastle, NSW Australia; 7 Manning Education Centre, Department of Rural Health, University of Newcastle, 69a High St, Taree, NSW Australia; 8 Central Coast Clinical School, University of Newcastle, Callaghan, NSW Australia; 9 Clinical Research Design and Statistical Services, Hunter Medical Research Institute, New Lambton Heights, NSW Australia

**Keywords:** Advance care directives, Dementia, Advance care planning, Junior doctors, Knowledge, Law

## Abstract

**Background:**

Junior medical doctors have a key role in discussions and decisions about treatment and end-of-life care for people with dementia in hospital. Little is known about junior doctors’ decision-making processes when treating people with dementia who have advance care directives (ACDs), or the factors that influence their decisions. To describe among junior doctors in relation to two hypothetical vignettes involving patients with dementia: (1) their legal compliance and decision-making process related to treatment decisions; (2) the factors influencing their clinical decision-making; and (3) the factors associated with accurate responses to one hypothetical vignette.

**Method:**

A cross-sectional survey of junior doctors, including trainees, interns, registrars and residents, on clinical rotation in five public hospitals located in one Australian state. The anonymous, investigator-developed survey was conducted between August 2018 and June 2019. Two hypothetical vignettes describing patients with dementia presenting to hospital with an ACD and either: (1) bacterial pneumonia; or (2) suspected stroke were presented in the survey. Participants were asked to indicate whether they would commence treatment, given the ACD instructions described in each vignette.

**Results:**

Overall, 116 junior doctors responded (35% consent rate). In Vignette 1, 58% of respondents (n = 67/116) selected the legally compliant option (i.e. not commence treatment). Participants who chose the legally compliant option perceived ‘following patient wishes’ (n = 32/67; 48%) and ‘legal requirements to follow ACDs’ (n = 32/67; 48%) as equally important reasons for complying with the ACD. The most common reason for not selecting the legally compliant option in Vignette 1 was the ‘*ACD is relevant in my decision-making process, but other factors are more relevant*’ (n = 14/37; 38%). In Vignette 2, 72% of respondents (n = 83/116) indicated they would commence treatment (i.e. not follow the ACD) and 18% (n = 21/116) selected they would not commence treatment. (i.e. follow the ACD). Similar reasons influenced participant decision-making in Vignette 2, a less legally certain scenario.

**Conclusions:**

There are critical gaps in junior doctors’ compliance with the law as it relates to the implementation of ACDs. Despite there being differences in relation to the legal answer and its certainty, clinical and ethical factors guided decision-making over and above the law in both vignettes. More education and training to guide junior doctors’ clinical decision-making and ensure compliance with the law is required.

## Background

It is estimated that 50 million people have dementia worldwide [1]. People with dementia are more likely to experience frequent or prolonged hospitalisations, and preventable complications and complex medical conditions place them at greater risk of adverse outcomes compared to those without a diagnosis of dementia [2–5]. In Australia, 71% of hospitalisations of people with a diagnosis of dementia are of the highest clinical complexity, compared to 16% of hospitalisations of people without a diagnosis of dementia [6]. An estimated one-third of older people will die with or from dementia; previous studies report that on average, people with dementia survive from three to ten years after their diagnosis [7–10].

As the number of people dying with or from dementia continues to increase, the need for high quality, person-centred end-of-life care is a recognised priority [11]. However, guidelines for care and treatment are still primarily consensus-based, and suboptimal care continues to be reported across a range of end-of-life areas [12–17]. While people with dementia may be less likely to receive aggressive care at the end of life, provision of palliative care interventions and symptom management remains inconsistent [13]. Limitations in cognitive capacity and the ability to communicate verbally underline the importance of interventions aimed at improving end-of-life care, such as advance care planning (ACP) [18]. ACP is a process whereby an individual discusses and documents their values, wishes and preferences about future health care, in case s/he later experiences periodic, temporary or permanent loss of decision-making capacity [19, 20]. ACP can include completion of a written document giving instructions about future care (i.e. an advance care directive [ACD]) and/or the legal appointment of someone to make decisions on a person’s behalf should they lose capacity (i.e. a substitute decision maker [SDM]) [21].

Failure to consistently offer, document and adhere to the wishes, values and preferences of people living with dementia represents a significant missed opportunity to improve the delivery of patient-centred end-of-life care [18, 22]. In Australia, the National Framework for Advance Care Planning recommends that ACP be a routine part of patient contact with health and aged care practitioners [23]. Given the unpredictable trajectory of dementia and cognitive decline, it is important that people with dementia are involved in ACP as early as possible to ensure they can be meaningfully involved in decision-making [18]. However, in a recent national audit of 2285 Australians aged 65 years or older [24, 25], only 30% of those who identified as living with dementia (n = 844) reported having a self-completed ACD, and 29% reported having an ACP document completed by a health professional or someone else on their behalf [26]. A recent systematic review demonstrated that ACP can be feasibly implemented with people with dementia; ACP has been associated with increased documentation, satisfaction with care and concordance between wishes and actual care, as well as decreased hospitalisation rates [27].

ACP instruments such as ACDs must be legally valid and accessible at various points of decision-making, be a reliable source of information, and be capable of being translated into practical clinical decisions [28]. State-specific legislation supporting ACDs result in different procedural requirements [29, 30] which can create uncertainty and confusion about the requirements of ACDs, their legal force, and the role of appointed SDMs. In New South Wales (NSW), an instructional ACD is recognised by common law, and the legal appointment of a SDM both by the person in advance of them losing capacity and by a tribunal is recognised [29, 31]. Variation in adherence to ACDs is attributed to an array of factors including doctors’ specialty and experience, their attitudes towards ACDs, and their knowledge of the law [30, 32]. While knowledge of the law related to ACDs can vary according to specialty, significant gaps remain [33–36]. Doctors often prioritise patient-related clinical and ethical considerations over the law when making medical decisions at the end of life [37]. Health professionals may also override instructions they perceive do not align with their perceptions of the ‘best interests’ of the patient, or struggle to translate vague preferences into specific clinical practice [38]. Health professionals may need reassurance that following ACP instructions will be legally and ethically supported.

The care of people with dementia in hospital is a key clinical skill for junior doctors. Despite their key role in discussions and decisions about treatment and end-of-life care for people with dementia, junior doctors report feeling underprepared for this role [39]. Commonly reported barriers relate to education and training, institutional policies and resources, as well as the additional complexity of determining prognosis and cognitive capacity among those diagnosed with dementia [39–41]. However, the few previous studies conducted with junior doctors have focused primarily on their skills related to symptom management and completion of limitation-of-care orders [39–43]. In comparison, the knowledge and perceptions of junior doctors regarding the implementation of ACP documentation are infrequently represented in research. The application of this knowledge in the context of dementia can be particularly challenging, given the likely greater vulnerability and added complexity of the patient. Therefore, the aim of this study was to describe among junior doctors in relation to two hypothetical vignettes involving patients diagnosed with dementia: (1) their legal compliance and decision-making process related to treatment decisions; (2) the factors influencing their clinical decision-making; and (3) the factors associated with accurate responses to one hypothetical vignette.

## Methods

### Design and setting

A cross-sectional survey was conducted at five hospitals located across two different local health districts in NSW, Australia. Four hospitals were in major cities, and one was located in an inner regional area.

### Sample

All junior medical doctors, including trainees, interns, registrars and residents, on clinical rotation at any of the participating hospitals at the time data collection occurred were eligible to participate. In the participating hospitals, junior staff rotate every 3–6 months.

### Recruitment

Potential participants were approached at scheduled training sessions, before or after ward rounds, or at change of rotation orientation days. Potential participants were given a verbal overview of the research by a member of the research team (also junior/senior doctors at participating hospital) and provided with a survey package that included a detailed information statement, a paper copy of the survey, and a reply paid envelope.

### Data collection

Data collection was carried out between August 2018 and June 2019. Consenting junior doctors were asked to complete a 63-item survey and return it by either handing it back to the research team after completing, placing it in a secure box in a hospital common room, or mailing it directly to the research team using a supplied reply-paid envelope. Return of a completed survey was taken as consent to participate. All participants were instructed that they could only complete the survey once.

### Measures

The survey included questions about resuscitation planning, advance care planning, substitute decision-making, demographic characteristics and clinical experience. (presented elsewhere) [44]. Only questions related to hypothetical vignettes are presented here. The development of the survey has been described elsewhere [44]. Briefly, items were derived from the literature on legal aspects of ACP, previous studies by the authorship team [36, 37, 44, 45] and legal and clinical expert opinions. A panel of behavioural scientists, lawyers, emergency physicians, general physicians and nurses refined the items. Items were refined based on the feedback of a pilot sample of five junior medical doctors.

For Vignette 1, participants were asked: ‘*As the treating physician, would you commence antibiotics?*’. Response options were: ‘yes’ (i.e. choose not to follow the ACD) or ‘no’ (i.e. choose to follow the ACD). The correct legal answer (determined in consultation with legal experts) is that antibiotics should not be provided and the ACD respected (i.e. response option ‘no’).

*Vignette 1.* Maria, a frail 75-year-old woman with advanced dementia, presents to hospital in a delirious state with bacterial pneumonia. Four years ago, when she was first diagnosed with dementia and still had capacity, Maria made an advance care directive. Maria's directive states that if she were to get a life-threatening infection, she does not want to receive antibiotics, but only be kept comfortable. Maria's designated substitute decision-maker provides her directive to you, her treating physician, but insists that she be treated for the infection. If given antibiotics, Maria is expected to fully recover. If antibiotics are withheld, it is likely that Maria will die.

For Vignette 2, participants were asked: ‘*As the treating physician, would you commence active treatment?*’ Response options were: ‘yes’ (i.e. choose not to follow the ACD) or ‘no’ (i.e. choose to follow the ACD). Vignette 2 was more legally, clinically and ethically complex, with sufficient uncertainty included to preclude a clear legal answer without further information or external support, such as from a hospital legal team or a court or tribunal making a ruling.

*Vignette 2.* Michael is 74 and has moderate dementia. He lives at home with his wife, Caroline, who is his designated substitute decision-maker, and are managing well with assistance from a home care package. Twenty years ago, while preparing for a physically challenging adventure, Michael completed an advance care directive stating that if he ever suffered a stroke, he wanted only to receive comfort measures. Michael presents to hospital with symptoms of a stroke. His arrival is within the timeframe to commence thrombolytic treatment, which recent evidence suggests is likely to result in return to pre-stroke levels of function. Caroline provides you with Michael's advance care directive, but asks that active treatment is provided.

For both vignettes, participants who responded ‘yes’ were then asked the reason for their choice from a list of five options: (1) ACD inconsistent with what is clinically indicated; (2) other factors more relevant to decisions; (3) SDM has legal authority to request treatment, even if in conflict with ACD; (4) don’t believe ACDs are appropriate to determine treatment; (5) ACD does not have legal effect. An open-ended ‘other’ option was also provided. Participants who responded ‘no’ to the question were asked the reason for their choice, including: (1) most important consideration is following the patient’s wishes; (2) most important is that the law requires me to follow the ACD; and (3) both of the above considerations are equally important. An open-ended ‘other’ option was again provided.

### Demographic characteristics, clinical experience and knowledge

Participants self-reported their gender, age category, Aboriginal or Torres Strait Islander status, religious affiliation, number of years’ experience as a doctor (post graduate year 1–4+), whether they were currently enrolled in a specialist training program (yes/no), whether they had ever participated in post-graduate courses or training about advance care planning (yes/no), and whether their medical degree was obtained in Australia or overseas. Six items explored knowledge of the legal validity of ACDs, legal authority of SDMs and treatment for patients without decision-making capacity [44].

### Statistical analysis

All statistical analyses were programmed using SAS v9.4 (SAS Institute, Cary, North Carolina, USA). Socio-demographic and clinical experience variables were summarised as frequencies and percentages for non-missing observations. Factors associated with legally compliant response to Vignette 1 were assessed using logistic regression. Participants not selecting the legally compliant option, including missing, were considered not legally compliant. Crude and multivariable odds ratios (OR) with 95% confidence intervals (CI) along with p-values from likelihood ratio tests were calculated. The Area Under the Curve Receiver Operating Characteristics curve (AUCROC) with 95%CI is provided. Characteristics identified at a *p* value < 0.05 were considered statistically significant.

### Ethics approval

Ethics approval was provided by the relevant Local Health District Human Research Ethics Committees (2018/ETH00209; 2018/STE00514).

## Results

### Sample

Overall, 116 of 328 distributed surveys were completed and returned (35% response rate). Table 1 presents the socio-demographics and clinical experiences of participants. The majority of the sample was female (n = 64, 56%), aged 20–29 years (n = 66, 58%) and had two or less years of post-graduate training (n = 59, 52%).

**Table 1:** Socio-demographic and clinical experience of participants (n = 116)

Variable	N	%
*Gender*		
Male	50	44
Female	64	56
Missing	2	
*Age*		
20–29	66	58
30 or over	48	42
Missing	2	
*Aboriginal or Torres strait islander*		
Yes	2	2
No	112	98
Missing	2	
*Medical degree obtained in Australia*		
Yes	86	76
No	27	24
Missing	3	
*Number of years’ experience*		
Post graduate year 1	18	16
Post graduate year 2	41	36
Post graduate year 3	9	8
Post graduate year 4 +	46	40
Missing	2	
*Enrolled in specialist training program*		
Yes	51	47
No	58	53
Missing	7	
*Post-graduate training (advance care planning)*		
Yes	14	12
No	98	88
Missing	4	
*Knowledge of legal validity of ACDs (6 items)*		
< 4 correct	86	75
≥ 4 correct	28	25
Missing	2	

### Vignette 1

Only 58% of respondents (n = 67/116) selected the legally compliant option (i.e., not commencing antibiotics). Table 2 presents the reasons given for selecting the legally compliant option. The most common reasons were ‘*Both of the above considerations are equally important’* and ‘*The most important consideration is following the patient’s wishes’*.

**Table 2:** Reasons selected by junior doctors for not commencing antibiotics (n = 67)*

Reasons	n	%
The most important consideration is following the patient’s wishes	32	48
The most important is that the law requires me to follow the ACD	1	1.5
Both of the above considerations are equally important	32	48

*Total does not sum to 67 due to missing responses

Almost a third (32%, n = 37/116) of respondents chose to ignore a legally binding ACD and commence antibiotics, while the remaining 10% were missing data. Table 3 presents reasons by respondents who chose to ignore the ACD; the most common reason was that the ‘*ACD is relevant in my decision-making process, but other factors are more relevant*’.

**Table 3:** Reasons selected by junior doctors for commencing antibiotics (n = 37)*

Reasons	n	%
Do not have to follow ACD because it is inconsistent with what is clinically indicated	4	11
ACD is relevant in my decision-making process, but other factors are more relevant	14	38
The SDM has legal authority to request life-sustaining treatment, even if it conflicts with the patient’s ACD	13	35
The ACD is not relevant to my decision-making because I don’t believe ACDs are appropriate to determine treatment	0	0
The ACD does not have legal effect	0	0

*Totals do not sum to 37 due to missing responses

### Factors associated with legally compliant response (not commencing antibiotics) to Vignette 1

After accounting for the demographic variables in the model, those with 4 or more correct responses to ‘knowledge of the legal validity of ACDs’ items (see Bryant et al. for published items) were 2.55 times more likely to answer Vignette 1 correctly, although this was not statistically significant (*p* = 0.08, 95% CI 0.88 to 7.36) (see Table 4).

**Table 4:** Crude and multivariate regression for legally compliant response (complete cases n = 106)

Variable and category	Odds ratio (95%CI)	*p* Value	Odds ratio (95%CI)	P- value	AUC ROC (95%CI)
*Gender*					
Female versus male	1.23 (0.58–2.60)	0.60	1.08 (0.46–2.54)	0.87	0.66 (0.56–0.77)
*Age*					
30 or more versus 20–29	0.72 (0.34–1.53)	0.40	0.57 (0.20–1.59)	0.28	
*Medical degree*					
Australia versus Overseas	1.82 (0.76–4.35)	0.18	1.51 (0.51–4.48)	0.46	
*Years of post-graduate experience*					
3 or more versus 2 or less	1.28 (0.60–2.70)	0.52	1.21 (0.38–3.88)	0.74	
*Enrolled in specialist training program*					
Yes versus No	1.74 (0.80–3.79)	0.16	1.90 (0.67–5.43)	0.23	
*Post-graduate training (ACP)*					
Yes versus No	1.87 (0.55–6.39)	0.32	1.65 (0.45–5.98)	0.45	
*Knowledge items*					
4+ versus < 4	2.07 (0.82–5.22)	0.12	2.55 (0.88–7.36)	0.08	

### Vignette 2

In response to the question about whether the physician would commence active treatment in Vignette 2, 72% of respondents (n = 83/116) selected yes and 18% (n = 21/116) selected no. Among those who responded ‘yes’, the most common reason selected was the ‘*ACD is relevant in my decision-making process, but other factors are more relevant*’ (Table 5).

**Table 5:** Reasons selected by junior doctors for commencing treatment (n = 83)*

Reasons	n	%
Do not have to follow ACD because it is inconsistent with what is clinically indicated	5	6
ACD is relevant in my decision-making process, but other factors are more relevant	37	45
The SDM has legal authority to request life-sustaining treatment, even if it conflicts with the patient’s ACD	15	18
The ACD is not relevant to my decision-making because I don’t believe ACDs are appropriate to determine treatment	1	1.2
The ACD does not have legal effect	1	1.2

*Totals do not sum to 83 due to missing responses

Among participants who responded ‘no’, the most common reason selected was ‘*Both considerations are equally important*’ (see Table 6).

**Table 6:** Reasons selected by junior doctors for not commencing treatment (n = 21)

Reasons	n	%
The most important consideration is following the patient’s wishes	6	29
The most important is that the law requires me to follow the ACD	1	5
Both of the above considerations are equally important	14	67

## Discussion

Knowledge of end-of-life law can potentially enhance decision-making processes, reduce legal risk and increase the likelihood of avoiding adverse patient and family outcomes [46]. However, our findings demonstrate there are gaps in the compliance of junior doctors with the law as it relates to the implementation of ACDs for individuals with dementia.

Vignette 1 provided an opportunity to test compliance with law using a hypothetical scenario in the context of dementia. As the patient had advanced dementia and was in a delirious state at the time, it is very unlikely that the patient would demonstrate sufficient capacity at the time of decision-making. Legal compliance was low among junior doctors. Limited knowledge about what constitutes a legally binding ACD and the circumstances in which ACDs should be followed has been previously reported in studies involving doctors, medical students and allied health professionals [37, 47, 48]. In a previous vignette study of decisions involving the use of ACDs by Australian and New Zealand doctors, agreement on treatment decisions varied according to the vignette complexity, ACD content, speciality and seniority of the doctor [35]. Doctors have identified subjective terminology, prognostic uncertainty, questionable validity and currency, family opposition, as well as time pressures as key barriers to ACD implementation [32, 38]. Notably, the majority of the sample was comprised of junior doctors (interns and residents), a group that have received less training and perhaps less exposure to these types of situations.

Vignette 2 reflects a clinical reality where time constraints mean that decisions may need to be made with incomplete information. The description implies the patient does not have capacity to make decisions as an ACD is presented, and it is the SDM requesting treatment. This conflict between ACD instructions and SDM wishes reflects a common dilemma faced by clinicians. Reflecting this uncertainty, only one fifth of participants reported they would follow the ACD. This suggests that in cases of doubt, the default preference of participants was to treat. Further, only one participant indicated they would provide treatment based on the assumption that ‘*The ACD does not have legal effect’*. This suggests that the legality of the ACD did not significantly contribute to participants’ decision-making process in this scenario. Furthermore, 18% of participants appeared to base their decision on a premise that is legally incorrect; that is, ‘*The SDM has legal authority to request life-sustaining treatment, even if it conflicts with the patient’s ACD’*. This is concerning, given that Australian general public report limited knowledge about the rights and responsibilities associated with the substitute decision-maker role [49]. This finding suggests that in addition to enhancing the legal literacy of junior doctors, educational resources and other interventions should also focus on increasing the awareness of the general community, particularly those living with dementia, regarding end-of-life decision-making and the law. This may increase the likelihood that people with dementia and their families are able to operationalise their legal rights, even in circumstances where doctor compliance is lacking [49, 50].

Despite differences between the two vignettes in relation to the legal answer and its certainty, the reasons participants gave for their decisions in both vignettes were similar. This suggests that factors that guide decision-making are stable, even when uncertainty about the law varies. Among those who would follow the ACD, following patient wishes or following both patient wishes and law were overwhelmingly selected as the reasons guiding these decisions. For the cohorts who indicated they would treat, the most selected basis for the decision in both vignettes was: ‘*ACD is relevant in my decision-making process, but other factors are more relevant*’, with the second one being that a *SDM can override an ACD*. Previously, physician adherence to ACDs has been found to be situation-specific, with time since completion of ACD, condition reversibility and family demands for care at odds with the ACD instructions reportedly influencing physician adherence [51]. Consistent with this, participants provided other potentially practical and clinical reasons for their decisions for Vignette 2. It is possible that adherence to the ACD may have been impacted by the time the ACD was made (20 years ago), and the considerable difference in outcomes and the emergence of effective treatments for stroke (e.g., thrombolysis) since time of completion. The ACD in Vignette 2 was also made by the patient in a completely different context (while preparing for a physically challenging adventure) when he presumably did not have a dementia diagnosis (compared to Vignette 1 who made the ACD more recently and at a time when her dementia diagnosis was known to her and she still had capacity).

Only one respondent in each vignette perceived the most important consideration to be the law alone, reflecting that legal considerations may not be the most significant consideration guiding junior doctors’ decision-making. These findings support emerging evidence that simply increasing knowledge of the law may not be sufficient to improve legal compliance [50]. In a previous vignette study examining the role of law in decisions about withholding and withdrawing life-sustaining treatment with specialists, the reasons given for complying with the law were similar among legally knowledgeable specialists and those who were not knowledgeable [37].

### Implications for practice, policy and future research

Together, these findings highlight that there are considerable gaps in compliance of junior doctors with the law. To increase the legal literacy of junior doctors, education and other skill-building interventions should be incorporated into post-graduate training and during clinical rotations, when experiential learning can develop transferable skills [39]. Junior doctors consider experiential learning an effective method for improving knowledge and skills in end-of-life care [39]. Additionally, acknowledgement of the clinical and ethical concerns of junior doctors which challenge compliance in the development of legal systems and processes relating to ACDs is required. This illustrates the potential benefit of medical-legal partnerships, when it comes to education and policy development initiatives in advance care planning [52, 53].

Our findings, particularly in relation to Vignette 2, also highlight the clinical and ethical complexity in cases where ACD's are not current and are occurring in the context of sometimes rapidly changing medical practice and diagnoses. Vague definitions of 'current' and 'apply to the clinical situation at hand' may also be a barrier to junior doctors following ACDs. Acknowledging the law is not as significant as other factors in clinical decision-making of junior medical doctors, at least in scenarios that explore adherence to ACDs, education resources to support clinical-decision making in relation to the recognition and management of people dying in hospital are critical [54]. Examples of initiatives introduced in Australian hospitals include the AMBER (Assessment; Management; Best practice; Engagement; Recovery uncertain) care bundle and the ‘Last Days of Life Toolkit’ [55].

In relation to vignette 2 where there is legal uncertainty, the appropriate course would be to engage internal legal advice processes which, in very rare cases, may be escalated to a court or tribunal (see, e.g. *Hunter and New England Area Health Service v A* (2009) 72 NSWLR 88 where the hospital applied to the New South Wales Supreme Court and received a determination that a ‘worksheet’ completed by A constituted an advance care directive that should be followed) [56]. Where there is genuine uncertainty, the courts have generally supported decisions made by clinicians in good faith and with good process (e.g., second opinion and engaging advice processes). There is a need for further work with larger samples of junior doctors and vignettes describing ethically and clinically complex scenarios.

### Limitations

Despite the study sample being drawn from five hospitals, the small sample size and response rate limits generalisability of the findings. However, these rates are similar to previous studies in this field [34–37, 40, 57]. Further, the hospitals were located in one Australian state. As each Australian jurisdiction has differing laws regarding ACDs, findings are also applicable to NSW only. However, previous research has shown that different legal frameworks across states does not always lead to different decisions about providing treatment [37]. The use of hypothetical scenarios, and the decisions made by participants in response to them, may not reflect the decisions that would be made in a real-life context where tangible rewards and consequences are likely to influence behaviour [58]. A qualitative component exploring junior medical doctors’ other reasons for legal compliance or non-compliance may have provided a more in-depth understanding of their clinical decision-making.

### Conclusion

There are critical gaps in compliance of junior doctors with the law as it relates to the implementation of ACDs. Although there was greater legal certainty about the requirement to follow the ACD in the first vignette, clinical and ethical factors guided decision-making over and above the law in both vignettes. More education and training on legal duties is needed to guide decision-making by junior doctors and ensure compliance with the law.

## Data Availability

The datasets used and/or analysed for this study are available from the corresponding author upon reasonable request.

## Abbreviations

ACP: Advance care planning
ACD: Advance care directive
SDM: Substitute decision maker
